# State-of-the-Art Genetic Modalities to Engineer Cyanobacteria for Sustainable Biosynthesis of Biofuel and Fine-Chemicals to Meet Bio–Economy Challenges

**DOI:** 10.3390/life9030054

**Published:** 2019-06-27

**Authors:** Aqib Zafar Khan, Muhammad Bilal, Shahid Mehmood, Ashutosh Sharma, Hafiz M. N. Iqbal

**Affiliations:** 1 State Key Laboratory of Microbial Metabolism, School of Life Sciences and Biotechnology, Shanghai Jiao Tong University, Shanghai 200240, China; 2 School of Life Science and Food Engineering, Huaiyin Institute of Technology, Huaian 223003, China; 3 Bio-X Institute, Key Laboratory for the Genetics of Developmental and Neuropsychiatric Disorders (Ministry of Education), Shanghai Jiao Tong University, Shanghai 200030, China; 4 Tecnologico de Monterrey, School of Engineering and Sciences, Campus Queretaro, Epigmenio Gonzalez 500, Queretaro CP 76130, Mexico; 5 Tecnologico de Monterrey, School of Engineering and Sciences, Campus Monterrey, Ave. Eugenio Garza Sada 2501, Monterrey CP 64849, N.L., Mexico

**Keywords:** cyanobacteria, metabolic engineering, commodity chemicals, genome-scale modeling, metabolic flux analysis, CRISPR/cas system

## Abstract

In recent years, metabolic engineering of microorganisms has attained much research interest to produce biofuels and industrially pertinent chemicals. Owing to the relatively fast growth rate, genetic malleability, and carbon neutral production process, cyanobacteria has been recognized as a specialized microorganism with a significant biotechnological perspective. Metabolically engineering cyanobacterial strains have shown great potential for the photosynthetic production of an array of valuable native or non-native chemicals and metabolites with profound agricultural and pharmaceutical significance using CO_2_ as a building block. In recent years, substantial improvements in developing and introducing novel and efficient genetic tools such as genome-scale modeling, high throughput omics analyses, synthetic/system biology tools, metabolic flux analysis and clustered regularly interspaced short palindromic repeats (CRISPR)-associated nuclease (CRISPR/cas) systems have been made for engineering cyanobacterial strains. Use of these tools and technologies has led to a greater understanding of the host metabolism, as well as endogenous and heterologous carbon regulation mechanisms which consequently results in the expansion of maximum productive ability and biochemical diversity. This review summarizes recent advances in engineering cyanobacteria to produce biofuel and industrially relevant fine chemicals of high interest. Moreover, the development and applications of cutting-edge toolboxes such as the CRISPR-cas9 system, synthetic biology, high-throughput “omics”, and metabolic flux analysis to engineer cyanobacteria for large-scale cultivation are also discussed.

## 1. Introduction

**Figure 1:**
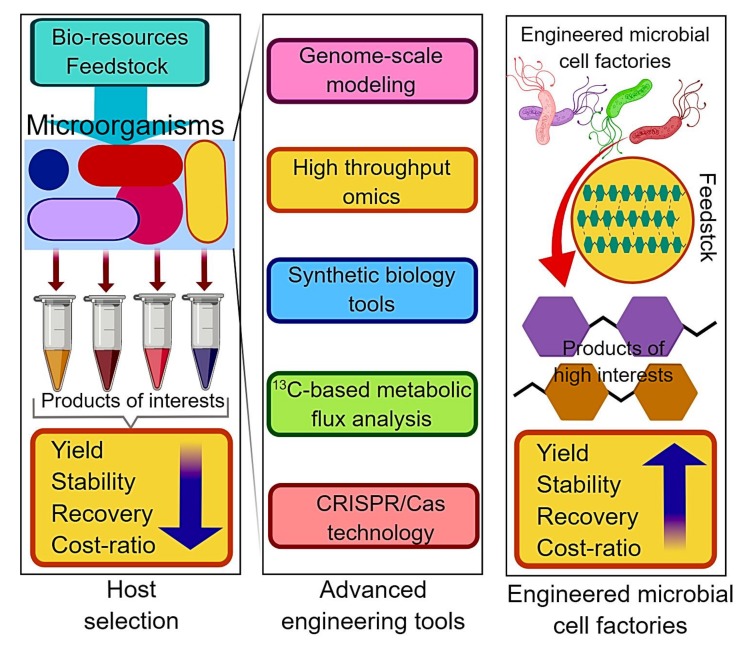
Advanced engineering tools to construct microbial cell factories for induced production of an array of value-added biochemicals.

Increasing apprehensions over energy and environmental issues are the key drivers for the development of renewable bio-based chemical products and fuels. Accelerating understanding of genomics and genetic manipulations enabled rapid improvement in the construction of microbial cell factories to produce an array of value-added bio-chemicals using diverse, sustainable bio-resources (Figure 1) [1,2,3]. Among the numerous microbial hosts, cyanobacteria have fascinated considerable research attention in the last few years as a promising platform for the sustainable and cost-effective production of industrially pertinent chemicals [4,5,6]. Following the green chemistry principles, cyanobacteria utilize atmospheric carbon dioxide (CO_2_) as a renewable feedstock and transform it into enormous valuable products, fuels, and commodity chemicals using sunlight as the potential energy source. The resultant carbon capturing and consumption technologies might exhibit a great perspective in alleviating the detrimental effects of raised CO_2_ levels if the technology scaled up to an industrial level. Notwithstanding the budding potential, several technical challenges need to be considered and address for rendering cyanobacteria-derived bioprocesses commercially feasible [2].

Cyanobacteria are photosynthetic prokaryotes inhabiting almost any environment that comprises water and possess the ability to grow in diverse environmental conditions [7]. These microbial hosts use photosynthesis and the Calvin–Benson cycle (CBC) for biomass production using merely CO_2_ and sunlight as the carbon and energy sources [4,8]. Engineering and rewiring the metabolic pathway of cyanobacteria offers the prospect for direct transformation of CO_2_ into value-added products. This approach could be beneficial over heterotrophic bio-production hosts necessitating plant-based fermentable sugars that have been intensively criticized due to their competition with humans and animals food supply. Interestingly, these photosynthetic prokaryotes offer distinctive advantages over plants as well as green algae and can capture solar energy more efficiently than plants. Also, they possess 9% conversion ability of the solar energy into biomass relative to higher plants transforming only 0.5–3% [9,10]. Cyanobacteria can be cultured in bioreactors in the arid or unfarmable land, which additionally diminishes the competition with food crops [11]. Nevertheless, the inevitability of significantly limited and expensive resources such as nitrogen and phosphorus inputs are a notable issue in these organisms as compared to plants or eukaryotic green algae [11,12]. Cultivation of the cyanobacteria in waste or salt water along with implementing nitrogen-fixing strains might be a partial solution to the shortcoming mentioned above [13].

After the elucidation of foremost metabolic engineering of a cyanobacterium for fuel ethanol production by Deng and Coleman [14], several succeeding research studies have revealed heterologous pathways expression to produce numerous compounds including alcohols, fatty acids, diols, and organic acids over the past twenty years (Table 1).

**Figure 2:**
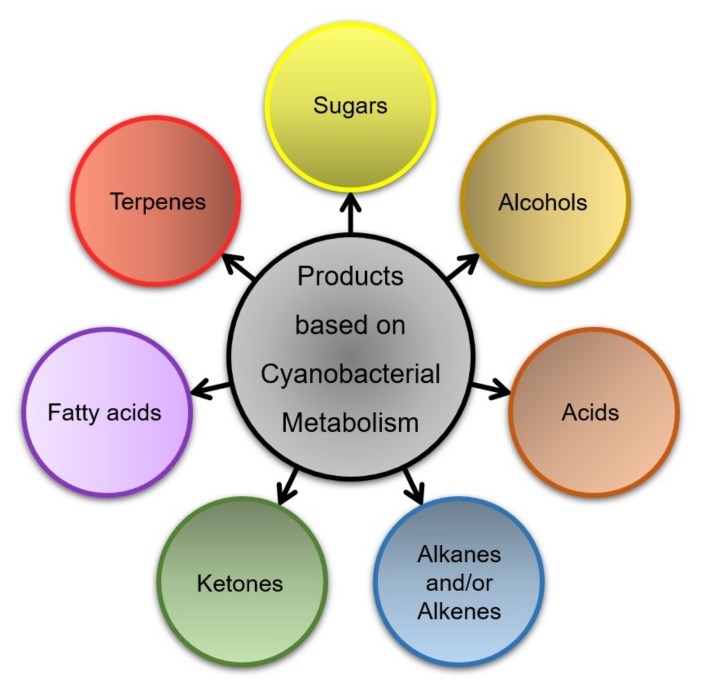
List of natural products metabolizes by cyanobacterial cells.

Besides, cyanobacteria have been documented to synthesize thousands of bioactive molecules [27,28]. Despite the long list of commodity chemicals produced by metabolically engineered strains, it is important to mention that the majority of these bioproducts are derived from the exploration of only a limited number of central metabolites. More recently, numerous sophisticated synthetic and metabolic engineering approaches have been introduced to improving the cyanobacterial genomic function for CO_2_ fixation and carbon rewiring flux to increase the ultimate photosynthetic product [29,30]. Recent advancements revealed that 50% of organic carbon had been successfully fixed during the photosynthetic reaction by engineered cyanobacteria [31]. While cyanobacterial systems can produce a great variety of industrially useful chemicals (Figure 2) in an energetically favorable way with a net negative atmospheric carbon contribution, they are considered as a promising platform for sustainable chemicals production [32]. Nevertheless, there are many limitations associated with the use of cyanobacteria in the production of chemicals at a commercial level. CO_2_ fixation is an intrinsically slow reaction occurred in the range of 1–4 reactions per second [33]. Furthermore, most of the heterologous pathways are initially designed and established on heterotrophic microbial candidates and subsequently transferred to photosynthetic prokaryotes, i.e., cyanobacteria [18]. Prominent dissimilarities in metabolism, transcription, and translation in these hosts make direct transfer often challenging and non-viable. Recent innovations in metabolic engineering led to the development of novel genetic tool such as the clustered regularly interspaced short palindromic repeats (CRISPR)-cas9 system, Cpf1 genome editing tool, high-throughput “omics”, and metabolic flux analysis. Adaptation to these tools has extended our capability to generate predictions and target-specific manipulations in cyanobacteria for the production of numerous chemicals at increasing titers [19,26,34,35,36,37].

This review encompasses the recent advances in engineering cyanobacteria for the production of biofuel and many other industrially relevant fine-chemicals. The development and applications of advanced technique and toolboxes for engineering cyanobacteria for their large-scale cultivation are also discussed.

## 2. Advanced Tools for Engineering Cyanobacteria

### 2.1. Genome-Scale Modeling

Genome-scale models (GSMs) is an important tool to delineate the entire metabolism of an organism by means of genetic data, and this technique plays a noteworthy role in the assessment and rewiring metabolic systems [19]. Without the appropriate knowledge and understanding of these approaches, it is highly challenging to scrutinize bottleneck steps, identifying the best-performing production host and optimizing the production of a target compound. In earlier studies, *E. coli* strain has been successfully tailored for the elevated biosynthesis of a wide variety of industrially relevant bioproducts such as ethanol, 1,4-butanediol, lactic acid, succinate, and lycopene by GSMs-directed engineering [38,39]. The contemporary development of two GSMs, namely iSyf715 [40] and iJB785 for *Synechococcus elongatus* PCC 7942 (hereafter referred to as *Synechococcus* 7942) results in better predictive power during the modifications and amendments to metabolism [40,41]. The model cyanobacterium *Synechocystis* PCC 6803 (hereafter referred to as *Synechocystis* 6803) exhibits a maximum doubling time of 7–10 h, whereas *Synechococcus* 2973 has shown the lowest doubling time of 1.9 h. This organism is closely related to *Synechococcus* 7942, with a total of 55 single nucleotide polymorphisms (SNPs) and a 188.6 kb inversion and a deletion of six open reading frames. Despite the slight differences, the growth rate for 2973 is 2.13 times higher as compared to 7942 under the optimal illumination conditions at 38 °C [42]. This rapid growth rate and current development of additional genetic tools render *Synechococcus* 2973 a promising host candidate for bioproduction. However, the development of *Synechococcus* 2973 as an attractive platform necessitates a deeper apprehension of its metabolic network and abilities. In this respect, Mueller et al. [43] established a genome-scale metabolic model, iSyu683, to develop *Synechococcus* 2973 as an ideal host candidate. For this, the experimental-based data were utilized to determine biomass composition and monitor carbon dioxide uptake for the strain. Applying this newly developed model, carbon uptake was recognized to be the major factor leading to the elevated growth rate of 2973 than 7942 [43]. For the said purpose, a genome-scale mapping model, i.e., *i*mSyu593, was constructed using the mapping model (*i*mSyn617) for *Synechocystis* sp. PCC 6803 (*Synechocystis* 6803). The fluxomics data showed complete conversion (>96%) of the assimilated carbon into biomass in *Synechococcus* 2973, while *Synechocystis* 6803 achieved only 86% conversion of the assimilated carbon [44]. Furthermore, four SNPs were recognized as potential contributors to modified kinetic parameters for metabolic enzymes. Continuous development of GSMs to evaluate predictions and manipulating model parameters by incorporating more experimental data will expand their usefulness in metabolic engineering.

### 2.2. High Throughput Omics

**Figure 3:**
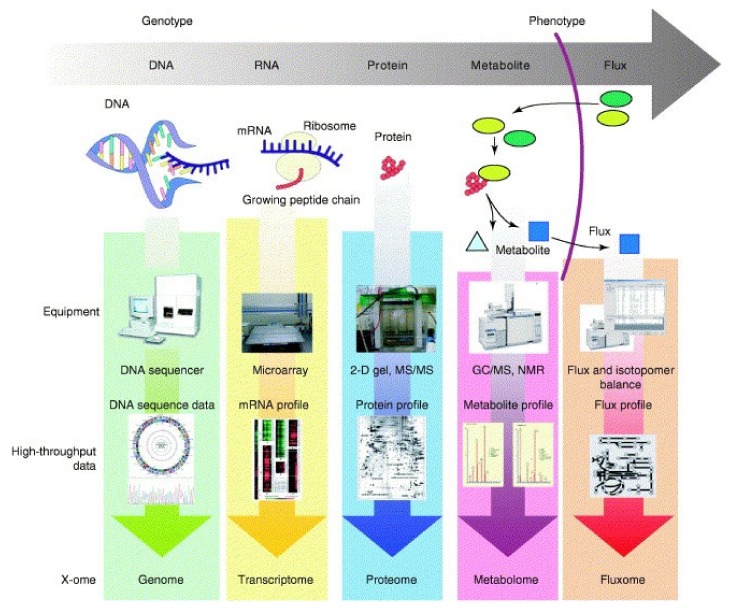
High-throughput omics research. Genomics advanced by the development of high-speed DNA sequencing is now accompanied by transcriptome profiling using DNA microarrays. Proteome profiling is joining the high-throughput race as 2D-gel electrophoresis combined with mass spectrography is advancing. Metabolome profiling is also rapidly advancing with the development of better GC/MS, LC/MS, and NMR technologies. Isotopomer profiling followed by challenging with isotopically labeled substrate allows determination of flux profiles in the cell (fluxome). Reprinted from Lee et al. [45], with permission from Elsevier. Copyright (2005) Elsevier Ltd.

In-depth understanding of various types of biological processes has been made possible by recent state-of-the-art via high-throughput experimental techniques. Additionally, these techniques are supported by bioinformatics, which has resulted in the rapid accumulation of a wide range of omics data at various levels (Figure 3) [45]. So far, a variety of analytical approaches have been developed and used for the genomic expression on a DNA, RNA, and proteomic level [46]. These approaches provide information inside the cells on their metabolism, external stimulus-response, and their development during the target molecules production [46]. Transcriptomic analysis of 6803’s cells under different abiotic stresses (i.e., temperature, light stress, depletion of inorganic carbon, nitrogen, iron, and phosphorus) has been carried out to identify small RNAs such as, CsiR1 NsiR4, PsiR1, IsaR1, and PsrR1 that are correlated to the cellular growth under stress conditions and photosynthesis [47]. The proteomic analysis demonstrates the metabolic burden of chemical production host strains [34]. For example, upregulation of proteins associated with the early stages of CO_2_ fixation results in 65% of fixed carbon into product formation in ethanol producing strains, whereas oxidative stress response related proteins were observed to be down-regulated in these strains [34].

### 2.3. Synthetic Biology Tools

**Figure 4:**
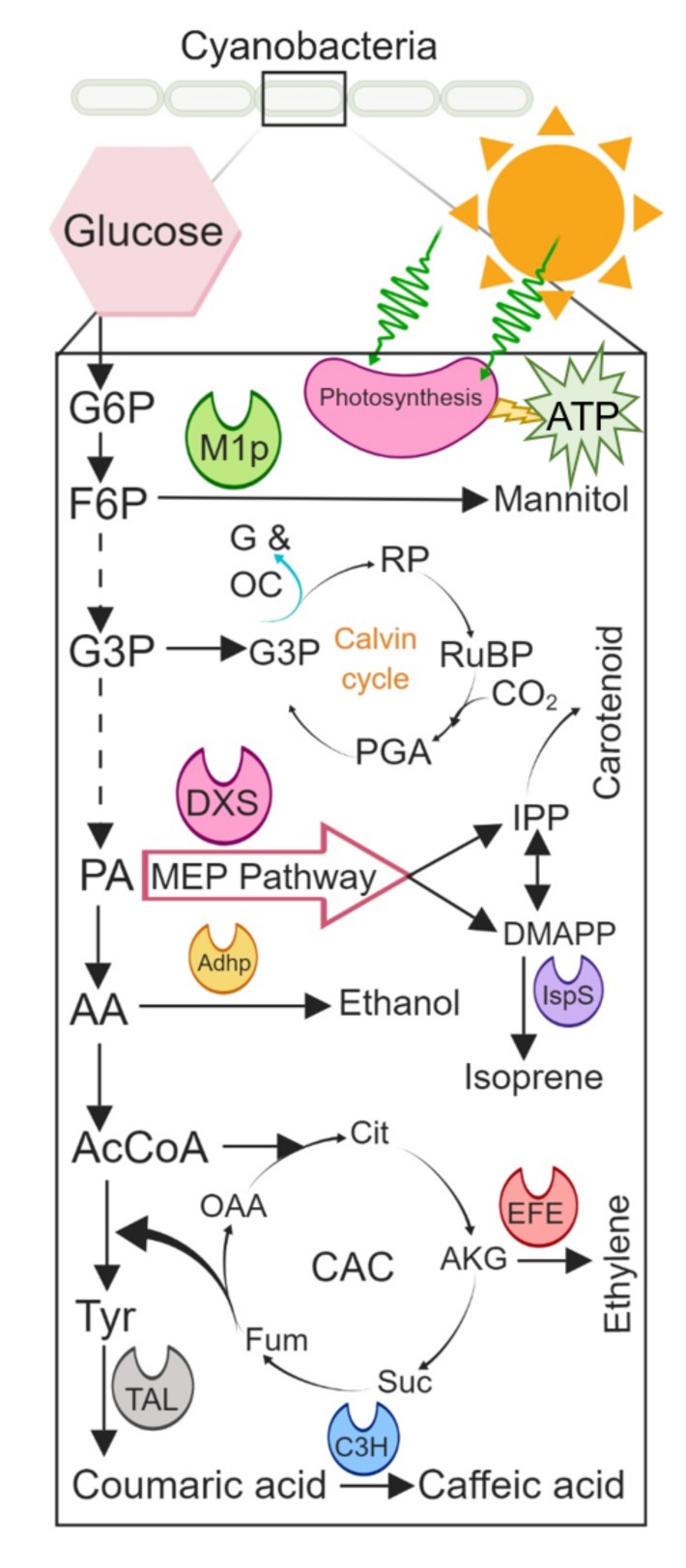
Graphical illustration of the metabolic pathway in cyanobacteria for the biosynthesis of value-added products. During the reduction of compounds into their target molecules, energy is required, which is supplied by photosystem in cyanobacteria where sunlight energy transferred H_2_O into energy-rich ATP and NADPH molecules. Abbreviations: G6P: glucose 6-phosphate; F6P: fructose 6-phosphate; G3P: glyceraldehyde 3-phosphate; RP: ribulose phosphate; RuBP—ribulose-1,5-bisphosphate; PGA: phosphoglycerate; M1p: mannitol-1-phosphatase; G and OC: glucose and other carbohydrates; PA: pyruvic acid; MEP: methylerythritol 4-phosphate; IPP: isopentenyl pyrophosphate; DMAPP: dimethylallyl pyrophosphate; DXS: 1-deoxy-d-xylulose-5-phosphate synthase; IspS: isoprene synthase; AA: acetaldehyde; Adhp: alcohol dehydrogenase; AcCoA: acetyl-clostridium coenzyme A (CoA); CAC: citric acid cycle; Cit: citrate; KAG: α-ketoglutarate; Suc: succinate; Fum: fumarate; OAA: oxaloacetate; Tyr: tyrosine; EFE: ethylene-forming enzyme; TAL: tyrosine ammonia-lyase; C3H: coumarate-3-hydroxylase.

The development of synthetic biology platforms is a powerful and eco-friendly approach for the dramatically improved biosynthesis of high-value compounds from sustainable carbon sources. The engineering of microbial-based biosynthetic pathways represents a newer approach to chemical synthesis with remarkable potential. A strapping set of synthetic biology tools, ranging from sites for expressing non-native genes, and tools for genetic manipulation and regulation of desired gene expression is necessary for constructing and utilizing a strain as a production host candidate. Over the years, several cyanobacterial-specified synthetic biology-derived approaches have been developed for target genes incorporation and regulating their expression in transcriptional as well as translational level due to the incompatibility of some *E. coli*-based genetic tools in cyanobacteria [48,49]. Similarly, a series of commercial biological products have been successfully developed through the design and construction of artificial biosynthesis pathways in cyanobacteria. Notably, six endogenous plasmids have been adapted to express non-native genes [50], and two additional sites exist on the genome for expressing non-native genes in 7002 [51]. Also, new methods for the regulation of gene expression at the transcriptional as well as translational level have also been established [51,52]. The gene expression can be controlled at the translational level in 7002 by riboswitches, which are regulatory segments of an mRNA that binds to a small molecule enabling an on/off translational response [51]. Markley et al. [49] tested a series of isopropyl *β*-D-1-thiogalactopyranoside (IPTG) inducible promoters based on *E. coli* promoters, namely PLlacO1 and Ptrc at the transcriptional level in 7002. They also designed and screened an ribosomal binding site (RBS) library to achieve the optimal dynamic range, and the best RBS and the promoter-integrated system showed a 48-fold dynamic range [49]. Apart from the IPTG-inducible systems, anhydrotetracycline-inducible promoters led to a 32-fold induction in 7002 [52]. Riboswitches and riboregulators are two suitable regulators designed for controlling expression at the post-transcriptional level and translational level (Figure 4) [53]. A theophylline-responsive riboswitch displays a dose-dependent (0.1–1.0 mM) response for the overexpression of gfp. Without theophylline, background fluorescence or inherent auto-fluorescence matches the wild-type, representing a tightly repressed inducible system accompanied by no leaky translation [53]. Similar to riboswitches, crRNA and taRNA riboregulator system also functions in controlling and expressing the genes in cyanobacteria. This riboregulator system resulted in a 13-fold enhancement in expressing a fluorescent protein along with notable induction in 6803 [54]. It is worth noting that riboregulator systems can also be employed to mimic the gene knockdowns effects and consequently creating them as desirable methods for the regulation of key genes related to critical cellular bioprocesses.

Gene cloning through plasmids shows higher expression in comparison with the direct gene integration into chromosomes of the host [55,56,57]. However, non-native genes revealed low expressions on the neutral site of chromosomes, which can be alleviated by means of constitutive promoters. This strategy enhances the expression to 2–4 times [57]. Rational promotor designing strategy was attempted to enhance the tunable gene expression by modifying –10 and –30 binding regions, and this approach helps to control the expression of a gene from high to low [55]. Recently, Wang et al. [22] compared a total of 17 natural and chimeric promoters and found that Ptrc promoter, featured with the *E. coli* σ70 consensus −35 and −10 elements, is the most suitable promoter in *Synechocystis* sp. PCC 6803 compared to the previously reported strong promoters, such as PcpcB and PpsbA, for the expression of ethylene forming enzyme (EFE) that limited the precursor supply and production of ethylene effects. No doubt, the IPTG-inducible promoter is promising and most commonly associated with some limitations that they require independent controlled gene induction. Therefore, nickel-tunable promoters, like *PnrsB*, improved the downstream gene expression up to 40-folds in contrast to the expression of the gene under the strong promoter *PpsbAII*. High light conditions cause down-regulation of *PnrsB* expression, which transfers the features to light-independent mediated gene expression in a diurnal setting for growth [58].

### 2.4. CRISPR/Cas Technology

**Figure 5:**
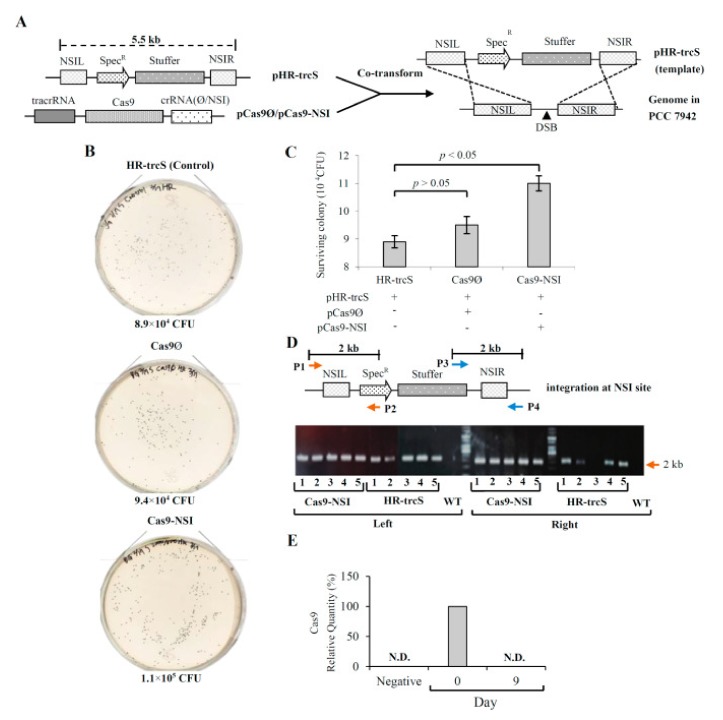
CRISPR/Cas9-mediated double strand break (DSB) promoted homologous recombination in PCC 7942. (**A**) Schematic illustration of the template plasmid pHR-trcS containing spectinomycin resistance (SpecR) gene, stuffer DNA and homology arms for NSI (NSIL and NSIR). (**B**) Photographs of spectinomycin-resistant colonies. (**C**) Quantification of spectinomycin-resistant colonies. (**D**) Colony PCR/electrophoresis to confirm precise integration. (**E**) Analysis of residual pCas9-NSI. Cells were singly transformed with 2000 ng pHR-trcS (HR-trcS group), co-transformed with 2000 ng pHR-trcS and 500 ng pCas9-NSI (Cas9-NSI group) or co-transformed with 2000 ng pHR-trcS and 500 ng pCas9Ø (Cas9Ø group). The cells were diluted 103-fold and streaked onto BG-11/agar plates containing 20 μg/mL spectinomycin. Five colonies were picked for colony PCR. Primer pairs P1/P2 and P3/P4 were designed to verify precise integration at the left and right junctions, respectively. Precise integration would give 2 kb amplicons after colony PCR. The residual pCas9-NSI plasmid was quantified by qPCR analysis of Cas9 gene in the cells. Reprinted from Li et al. [60], with permission from Elsevier. Copyright (2016) International Metabolic Engineering Society. Published by Elsevier Inc.

At contemporary, CRISPR technology appears as a promising approach to increase the speed and efficiency of genomic manipulations in cyanobacteria [59,60]. This approach allows marker-less genome editing, instantaneous modification of numerous genes, as well as the transcriptional regulation of multiple genes in the significantly shorter timeframe, which is greatly useful for tailoring cyanobacterial strains [61]. CRISPR system can generate highly efficient homologous recombination Cas9 in 7942 by increasing double-stranded breaks at the target integration locations that allow for reduced homology arms and lesser template DNA concentrations in transformation [60]. Though genetic stability is demonstrated as a major problem in engineering cyanobacteria [30,62], however, the CRISPR-Cas9 system allows scarless and iterative based accelerated stable recombination [60]. Inactivation of *glgC* in a succinate-producing 7942 by CRISPR-Cas9 approach validates its capability to induce practical modifications in metabolically engineered strains (Figure 5) [60]. Also, CRISPRi has also been effectively explored to knock down numerous target genes in the identical succinate-synthesizing microbial hosts [36]. The development of a CRISPR/cas9 system has also been studied in 2973 [63]. Preliminary application of the *Streptococcus pyogenes*-based Cas9 enzyme exerted toxic effects in the strain. Nevertheless, the use of a transient expression vector for this enzyme accomplishes marker-less manipulations/editing in all the tested bacterial mutants [63]. Furthermore, a cas9 expression associated with antibiotic resistance can be relieved by cultivating the strains in antibiotic lacking conditions [63]. Overall, this approach provides a milestone inducing genetic amendments in 2973, and further assists in extending this to several other cyanobacterial host candidates. The simplicity and easiness with which this system is malleable to cyanobacterial strains are highly encouraging. However, CRISPR technology has not been widely used in cyanobacteria because of the apparent toxicity of the Cas9 nuclease in these photosynthetic organisms. To overcome this obstacle, Ungerer and Pakrasi, [35] developed a precise and highly efficient tool for creating numerous markerless modifications in cyanobacteria using CRISPR technology and a novel RNA-directed dsDNA nuclease, Cpf1 that has been determined to be nontoxic to cyanobacteria. Cpf1 is a dual nuclease that displays specific ribonuclease activity cleaving 36 bp repeat of the pre-CRISPR RNAs (pre-crRNA) nucleotides upstream of a hairpin in an Mg^2+^ dependent manner. The mature crRNA then directs cpf1 to its DNA target where its nuclease activity induces a 5 bp double-stranded break. The cpf1 system is considered more cost-effective in synthetic biology as it uses only a 42 nt RNA component, which is considerably cheaper to synthesize than the >100 nt gRNA required by *cas9* systems. The markerless nature of the cpf1 genome editing tool enables complex genome alteration that was not possible with already available technologies. Scaling up the number and efficacy of modifications to competing for those of *E. coli* and *S. cerevisiae* will surely be the forward step in facilitating the construction of cyanobacteria as highly promising bio-factories.

### 2.5. ^13^C-Based Metabolic Flux Analysis

In recent years, measuring intracellular metabolism has increasingly led to important insights and comprehensions in biotechnology, biochemical engineering, and biomedical research. In this context, ^13^C-tracer analysis has been regarded as a promising and time-efficient tool to unveil relative pathway activities and extracellular or intracellular metabolite levels. ^13^C metabolic flux analysis (^13^C MFA) is used to calculate metabolites flux more precisely and helps to understand the biosynthetic pathway, which can be further manipulated through metabolic engineering to get desired production [49]. Lack of extensive experimental information about metabolic flux in cyanobacteria is a major hindrance in developing metabolic engineering-based novel tools, in particular, genome-scale model validation [64]. In cyanobacteria, ^13^C labeling is essentially restricted to labeled C1 compounds, CO_2_ and bicarbonate; however, labeling of heterotrophic carbon sources, such as glucose or glycerol is of profound importance to get insightful and robust information from metabolic flux analysis [37]. The development of comprehensive procedures for sample collection, isotopic labeling, and mass spectrometry examination has led to a new method called isotopically nonstationary ^13^C MFA (INST-MFA). This method can be potentially applied to cyanobacteria to overcome the autotrophic growth incompatibility challenge with the steady-state prerequisite of ^13^C MFA [64,65]. In a metabolically engineered 7942 strain for hyper-production isobutyraldehyde, INST-MFA recognized a bottleneck at pyruvate kinase and allows target-specific engineering by overexpressing pyruvate kinase encoding *pyk* or pathway bypass to address the drawback [66]. The metabolic flux of labeled NaH^13^CO_3_ as an inorganic carbon was employed to scrutinize changes in carbon partitioning in 6803 and *nrt*ABCD knockout mutants under nitrogen-depleted conditions [37]. INST-MFA analysis revealed an elevated flux level of glycogen pathway and anaplerotic reactions in *nrt*ABCD knockout derivatives than that to native strains [37]. Also, mutants also necessitated higher amounts of levels NADPH and ATP demonstrating the consumption of ATP without regenerating NADPH in the Calvin–Benson (CB) cycle [37].

## 3. Engineering Cyanobacteria for Environmental Stress Resistance

Cyanobacterial cell factories display much potential due to their survival against diverse environmental stresses. These environmental stresses were mainly concentrated on four aspects:

(1) Seawater or industrial wastewater is utilized for larger-scale cyanobacterial culturing than that of sterilized fresh water because of economic and environmental costs, where the presence of heavy metals, salts, and many other potential toxins will interrupt the normal cellular growth and metabolism of cyanobacterial cell factories.
(2) Application of selective conditions for bio-contamination control such as extreme low/high pH and elevated NaCl concentration can also impede the normal growth and metabolism.
(3) The accumulation of toxic intermediates products and metabolites in the cultivation system.
(4) Temperature or light intensities in the real environmental situations will be controlled in a rhythm, with peak levels that are too extreme for cyanobacterial strains to acclimate.

The metabolic capacities optimized at the lab scale will be disturbed and declined under the imbalanced intracellular as well as extracellular environmental conditions. Therefore, these factors intended to be retained in an optimal fashion to achieve an optimum titer of target bio-products. For instance, high pH has recently been described as playing a key contribution in the biosynthesis and recovery of pyruvate and succinate by cyanobacterial cell factories [67]. Though, appropriately designing process-engineering approaches might alleviate abiotic stresses by maintaining stabilized cultivation conditions, the greater stress-tolerances capacity of the cyanobacteria is of paramount significance to generate robust photosynthetic manufacturing in outdoor environments. The selection of promising and more suitable cyanobacterial chassis can also be considered as another option. Nevertheless, lack of clear genetic backgrounds and widespread genetic engineering tools are the major hindrances, and recent synthetic biology and metabolic engineering-based concerted efforts have only been devoted on model cyanobacterial organisms, such as *Synechocystis* 6803, *Synechococcus* 7942, and *Synechococcus* 7002 owing to the easy genetic modifications [31]. In this context, designing and engineering newer tools and technologies of broader sophistication could enhance metabolic physiology and robustness in cyanobacteria.

### 3.1. Introducing Heterologous Stress Tolerance Proteins

Extremophilic bacteria can grow, reproduce, and survive in severe environmental situations. Identification and installation of tolerance-relevant genes from extremophiles appear as a promising approach to improve the metabolic physiology and cellular robustness of cyanobacteria [5]. Following the introduction of molecular devices from a halotolerant cyanobacterium, the salinity stress tolerance of freshwater cyanobacterial strains has been effectively improved. Numerous salts-tolerance pertinent genes have recently been identified and characterized in a halotolerant cyanobacterium *Aphanothece halophytica*, which can grow and survive in hyper-osmotic settings in the presence of 3 M NaCl salt [68]. Waditee et al. [69] isolated a *nhaP* gene from *A. halophytica* and introduced into a cyanobacterial strain *S. elongates* PCC7942. Results showed that the resulting recombinant strain was able to grow and survive in the presence of 0.5 M NaCl [69]. In addition, the PCC7942 strain showed an optimized growth performance in seawater by the co-expression of *nhaP* (encoding a Na+/H+ antiporter) with *katE* (*E. coli*-sourced catalase gene). However, the salt tolerating capacity was not further enhanced [69]. The production and accumulation of glycine betaine are regarded as another indispensable way for *A. halophytica* to tolerate high osmotic stress imposed by high concentrations of salt. For example, incorporation of the glycine betaine encoding devices or system to two freshwater cyanobacteria *Anabaena sp.* PCC7120 and *A. doliolum* resulted in the production of glycine betaine, which in turn effectively enhanced salt tolerance [70]. Improvement in system biology and simulation of metabolic network’s tools enable more effective identification and prediction of functional devices related to the resistance under harsh environmental conditions. 

### 3.2. Enhancing Cyanobacterial Robustness by Overexpressing Heat Shock Proteins

Heat shock proteins (HSPs) are a family of proteins that constitute the most significant components for a bacterial stress-related response. Primarily, these proteins are induced under different kinds of environmental stresses preventing protein aggregation/denaturation and facilitate the proteins folding. Overexpression of *groESL* enhanced the cellular physiological resistances against various stresses enabling the growth of the nitrogen-fixing cyanobacterium *Anabaena* sp. PCC7120 strain at 42 °C and 50% NaCl (0.15 M) [71]. Similarly, the overexpression of *clpB1* in *Synechocystis* 6803 increased cell survival rates up to 20-folds under rapid heat shock conditions by preventing protein disaggregation [72]. The outdoor cultivation malleability of *Synechococcus* 7942 was significantly improved by the overexpression of a small HSP-encoding *hspA* gene, and the resulted engineered strain exhibited continuous growth in seawater containing closed photo-bioreactors under outdoor settings of temperature and light intensities, and high salt [73]. Table 2 displays the growth of various engineered cyanobacterial strains under different stress conditions and growth system.

## 4. Engineering Cyanobacteria for Biofuel and Fine Chemicals Production

### 4.1. Cyanobacteria—Biofuel

In recent years, ethanol production by the biological method has attracted significant research attention. Previously, a two-step biosynthetic route comprising a collection of plant-based feedstocks followed by its transformation to fuels by microbial fermentation is used [78]. This indirect biosynthetic approach seems ineffective in converting the recalcitrant biomasses to biofuels, and therefore, accelerating interest has been diverted to use photosynthetic prokaryotes for direct conversion of carbon dioxide to fuels. Though native cyanobacterial strains are naturally unable to be a high ethanol titer producer. Thus, it is of significance to boost the biosynthesis efficacy of these strains to realize an economically feasible level [79]. Metabolic engineering and synthetic biology-based approaches, including the substitution of natural enzymes with proficient alternatives, enzyme engineering/optimization, tailoring regulators or cofactors, installing innovative pathways, and manipulating photosynthesis and carbon fixation have now made it possible to efficiently rewirite pathways in the microbial cells to achieve higher yields [80]. For the first time, Deng and Coleman [14] developed a novel pathway for enhanced ethanol production by introducing *pdc* and *adh* genes encoding pyruvate decarboxylase and encoding and alcohol dehydrogenase II, respectively, into a *Synechococcus* 7942 from the *Zymomonas mobilis* bacterium. Results showed that the resulting engineered cyanobacterial strain accumulated a substantial amount of ethanol in the fermented culture. Subsequently, Gao et al. [18] designed a genetically tailored *Synechocystis* 6803 strain with considerably elevated ethanol biosynthesis efficiency of 5.5 g/L. The strategies included introduced of an additional copy of pyruvate decarboxylase, chromosomal overexpression of endogenous alcohol dehydrogenase, and inactivation of the poly-*β*-hydroxybutyrate biosynthetic pathway. Also, the influences of various cultivating conditions such as tap water, metal ions, and anoxic airing on ethanol biosynthesis were also investigated. Considering the simple cultivation requirements accompanied by a high-level ethanol production efficiency indicates cyanobacteria to be a promising platform for bioconversion of CO_2_ and solar energy into many values added bioproducts.

### 4.2. Cyanobacteria—Isobutanol and 1-Butanol

In contrast to ethanol, Isobutanol and 1-butanol are considered as superior gasoline alternatives owing to their higher energy density, less volatility, and less corrosiveness [81]. Notably, isobutanol display extensive applications as an organic solvent, and fuel additive, and its ester derivatives are widely used as polymer plasticizers [82]. Direct photosynthetic biosynthesis of isobutanol in cyanobacterial strains is also achievable using metabolic engineering and synthetic biology approaches. For example, the construction of a non-native isobutanol biosynthesis pathway in *Synechococcus* 7942 results in the accumulation of up to 450 and 1100 mg/L isobutanol and isobutyraldehyde, respectively [83]. Li et al. [84] constructed a glycogen synthase mutant of *Synechococcus* 7942 to increase the flow of carbon towards isobutanol. Inactivation of *glgC* gene encoding glucose-1-phosphate adenylyltransferase led to growth retardation under high light intensity in the absence of the isobutanol pathway. Whereas the introduction and overexpression of the iso-butanol pathway significantly (22% to 52%) enhanced the isobutanol production. Protein engineering of α-ketoisovalerate decarboxylase was utilized to enhance catalytic activity and iso-butanol production in *Synechocystis* 6803. A single replacement of either Ser286 to threonine or Val461 to isoleucine significantly improved the α-ketoisovalerate decarboxylase activity resulting in increased isobutanol production. Engineered strain with the combined modification V461I/S286T displayed 2.4 times improvement of isobutanol [85]. Sun et al. [86] described the identification and functional characterization of a novel 124 nt sRNA Ncl1460 associated with 1-butanol tolerance in *Synechocystis* 6803. Expression analysis revealed that Ncl1460 was a negative regulator of *slr0847* and *slr0848* operon responsible for coenzyme A biosynthesis through promoter-driven transcriptional silencing mechanisms. Furthermore, a quantitative proteomics analysis demonstrated that CoaR regulated tolerance to 1-butanol by clostridium coenzyme A (CoA) biosynthesis down-regulation, resulting in a diminished fatty acid and energy metabolism. An engineered CoA-dependent pathway was introduced, for the first time, into *Synechococcus* 7942 for the direct photosynthetic conversion of CO_2_ to butyrate. Two CoA removal strategies were then separately incorporated to the modified CoA dependent pathway to increase the titer of butyrate. The best butyrate producing strain constructed resulted in an observed butyrate titer of 750 mg/L and a cumulative titer of 1.1 g/L [87]. Higo and Ehira, [88] created *Anabaena* strains expressing enzymes of the 1-butanol synthetic pathway from the anaerobe *Clostridium acetobutylicum*. A strain with overexpression of a highly oxygen-sensitive Bcd/EtfAB complex was able to produce 1-butanol even under photosynthetic conditions. Moreover, the 1-butanol biosynthesis per heterocyst cell of a butanol-producing *Anabaena* strain was five-fold higher than that per cell of unicellular cyanobacterium with an identical set of 1-butanol biosynthetic pathway genes.

### 4.3. Cyanobacteria—Hydrogen

Hydrogen gas is generated as a byproduct of nitrogen fixation under nitrogen-depleted cultivation environment in filamentous cyanobacteria. It is described as one of the most preferred substitutes for traditional fossil fuel-based energy sources owing to good efficiency, environmentally clean, and renewability. Cyanobacteria are highly promising microorganisms for hydrogen production. Hydrogen production by cyanobacterial strains is commercially more justified and feasible than the conventional chemical and photoelectrical methods [89]. The production of hydrogen has been investigated in numerous photosynthetic cyanobacterial strains, and at least 14 cyanobacteria genera are known producers of hydrogen under diverse growth environments [90]. Cyanobacterial strains possess two distinct kinds of hydrogenases: oxygen oxidizing uptake hydrogenases and reversible hydrogenases that can have a role in both take up as well as hydrogen production [91]. In some cyanobacteria, the hydrogen biosynthesis efficacy is restricted by the extreme oxygen sensitivity of hydrogenases and the tendency for [NiFe] hydrogenases to thermodynamically favor hydrogen uptake [92]. Elevated hydrogen production can be accomplished by blocking or inactivating pathways that compete with hydrogenases for reductant consumption. Inactivating NADH utilizing *ldhA* gene in *Synechococcus* 7002 led to a considerable improvement in the NADH/NAD+ ratio accompanied by a five-fold elevation in hydrogen production [93]. Similarly, Ducat et al. [94] reported an improved hydrogen titer in *Synechococcus* 7942 by heterologous expression of exogenous [FeFe] hydrogenases from *Clostridium acetobutylicum*. Engineering cyanobacteria by redirecting glycogen catabolism through the pentose phosphate pathway (PPP) has shown a substantial improvement in the accumulation of intracellular NADPH and therefore increased the hydrogen production. In contrast to the wild-type strain, a 2.3- and three-fold increase in hydrogen level was obtained noted by Kumaraswamy et al. [95] in the engineered *Synechococcus* 7002 strain with inactivated *gap*1 gene accompanied by the overexpression of NAD^+^-dependent glyceraldehyde-3-phosphate (GAPDH-1). More recently, Maswanna et al. [96] immobilized *Tetraspora* CU2551 cells in alginate matrix by an entrapment approach to investigate the reduced O_2_ exposure to the cells. Under optimized immobilization conditions of 4.0% alginate concentration, cell concentration 0.125 mg dry cells/mL alginate and bead size of 2.8–3.35 mm, the hydrogen production was significantly improved in the culture medium reaching 0.3 mL H_2_/mL medium. Interestingly, this titer was found to be 6 times higher than cells suspension, 2–10 times higher than other green algae, and 10–50 times higher when compared with cyanobacteria. It can be inferred that support-immobilized cells constitute a promising approach for enhanced production of photobiological hydrogen in the photosynthetic organisms.

### 4.4. Cyanobacteria—1,3-Propanediol

1,3-propanediol (1,3-PDO) is an important chemical with wide applications in the manufacturing of copolymer, solvents, paints, and anti-freezing agents [97]. Low productivity titers and complicated chemical procedures for 1,3-PDO production encourage the development and construction of bio-based methods for 1,3-PDO synthesis. Hirokawa et al. [98] constructed a biosynthetic route in *S. elongatus* PCC 7942 to produce 1,3-propanediol. In this metabolic pathway, dihydroxyacetone phosphate (DHAP) is initially transformed to glycerol though glycerol-3-phosphate (GAP). The resulting glycerol then gives rise to the synthesis of 1,3-PDO via 3-hydroxypropionaldehyde (3-HPA). A maximum titer of 3.79 mM was achieved 1,3-PDO after under optimized cultivation conditions of 14 days by the final engineered *strain.* The same research group further enhanced the yields of 1,3-PDO by disrupting genes screening via *in-silico* simulation. At first, the cellular metabolic flux distribution (MFD) in engineered 7942 strain was predicted by applying a stoichiometric metabolic flux balance analysis (FBA) model. Then, a genome-scale model of 7942 previously developed by Knoop was manipulated by incorporating an artificial 1,3-PDO biosynthetic pathway. Afterward, the MFD was employed for simulating gene disruption, and the influence of gene disruption was determined in the 1,3-PDO synthesizing strain. Results showed that the *ndhF1*-disrupted engineered strain displayed the elevated titer of 4.44 mM 1,3-PDO after incubating for 20 days [99].

### 4.5. Cyanobacteria—Fatty Acid and Hydrocarbons Biosynthesis

Fatty acids are a promising target for biosynthesis as they can be easily transformed into diesel-like compounds. During the lipid biosynthesis, hydrocarbon chains are extended following condensing acyl-acyl carrier protein (ACP) with CoA-bound carbon units, and the discontinuity of acyl-ACP by mean of a thioesterase produces free fatty acids (FFA). Several photosynthetic cyanobacterial strains such as 6803, 7942, and 7002 have been tailored to produce fatty acids [100,101]. Liu et al. [100] achieved an optimized biosynthesis of FFA in 6803 by eliminating alternative carbon sinks in combination with diverting carbon flux towards the fatty acid route, and resultantly, the engineered strain produced an elevated titer and productivity of 197 mg/L and 0.4 mg/L/h, respectively. Numerous cyanobacterial species have a natural tendency to produce small amounts of fatty hydrocarbons [4]. Tan et al. [102] carried out the production of fatty alcohols by a genetically engineered PCC6803 strain with a final titer of about 0.2 mg/L following the overexpression of fatty acyl-CoA reductase enzyme. As biosynthesis of hydrocarbon is a natural ability of many cyanobacterial strains, expressing alkane production genes has been demonstrated a promising strategy for improving the alkane titer. Nevertheless, both hydrocarbon and fatty alcohol titers were found to be rather lower than that to an enhanced level of fatty acids. Yoshino et al. [103] synthesized 4 µg/g DCW of heptadecane along with small amounts of unsaturated hydrocarbons (i.e., 1-non-adecene, 1,14-nonadecadiene) by the overexpression of *ado* and *aar* genes in *Synechococcus sp*. NKBG15041c.

### 4.6. Engineering Cynobacteria for Organic Acids Biosynthesis

#### 4.6.1. Cyanobacteria—Lactic Acid

L-Lactic acid is a high-value chemical, widely employed in the pharmaceutical and food industries and can be synthesized from pyruvate in a single enzymatically catalyzed step by NADH-dependent lactate dehydrogenase (Ldh). Recently, Angermayr and coworkers [84] explored a combination of four strategies including augmented *ldh* gene amount, a mutation in *B. subtilis* Ldh for NADPH utilization, blockage of phosphoenolpyruvate (PEP) competing reactions, and overexpression of pyruvate kinase enzyme for improved production of lactate. Results revealed that the resulting engineered 6803 strain produced a high titer and productivity of 840 mg/L and 22 mg/g DCW/h. Under the nitrogen-depleted environment, removal of stored glycogen led to a two-fold increase in L-lactate titer, while poly-3HB elimination exhibited no identical effect on lactate production [104]. More recently, Hirokawa et al. [98] tailored *S.* *elongatus* PCC 7942 cyanobacterial strain that possesses a potential ability to produce lactate directly from CO_2_ by using dihydroxyacetone phosphate (DHAP). A *mgsA gene* encoding methylglyoxal synthase from *E.* coli was integrated into 7942 strain for efficiently converting DHAP to methylglyoxal. In addition to this, genes encoding lactate/H^+^ symporter and putative glyoxalase I, II were also incorporated for enhanced lactate biosynthesis. Results evidenced that the engineered cyanobacteria strain with newly installed lactate-synthesizing pathway accumulated the highest titer of 13.7 mM lactate after 24-days incubation.

#### 4.6.2. Cyanobacteria—3-Hydroxpropionate

3-Hydroxypropionic acid (3HP) is an attractive molecule which can be used as an excellent precursor to manufacture a variety of high-value products such as acrylamide, acrylonitrile, acrylic acid, bioplastics, malonic acid, 1,3-propanediol, and many homo- and heteropolymers [105] owing to the presence of two functional groups. The United States Department of Energy (DOE) recognizes 3-HP as one of the top 12 building block chemicals in the list issued in 2004 [106]. Two biosynthetic pathways, including malonyl-CoA dependent pathway and *β*-alanine dependent pathway have been reported in 7942 to produce 3HP [107]. In the malonyl-CoA dependent pathway, 3HP is originated by the reduction of malonyl-CoA is via intermediate malonate semialdehyde. The *β*-alanine dependent pathway starts with PEP carboxylation to produce oxaloacetate, which is subsequently transaminated into aspartate. The decarboxylation of the resulting aspartate yields *β*-alanine. *β*-alanine is transaminated to malonate semialdehyde and finally reduced to 3-HP [107]. Individually overexpressing genes of malonyl-CoA dependent pathway in S. elongates strain resulted in a final titer of 665 mg/L 3HP, whereas the same strain *β*-produced up to 186 mg/L 3Hp following the expression of alanine dependent pathway indicating the practicality of transforming CO_2_ into 3HP by engineered cyanobacterial strains.

### 4.7. Engineering Cyanobacteria for Carbohydrates/Sugars Biosynthesis

In comparison to heterotrophic hosts (i.e., *E. coli*, and yeast), native cyanobacteria are usually less tolerant to chemicals. Thus, genetically engineered cyanobacterial strains are used to synthesize soluble sugars, which are less or not toxic. Glycogen, naturally accumulated in cyanobacterial strains for carbon storage, is an important and promising feedstock material for producing biofuel [108]. Aikawa and coworkers [20] optimized a range of conditions such as CO_2_ concentration, salinity, and light intensity for elevating the glycogen accumulation in 7002. The strain produced a high titer 3.5 g/L of glycogen representing corresponding at the productivity of 0.5 g/L/d under the optimized conditions. More recently, Badary et al. [109] investigated the glycogen biosynthesis by a cyanobacterial strain NKBG15041c using various growth conditions. Up to a yield of 399 μg/mL/OD_730,_ glycogen was recorded by cultivating NKBG15041c cells in nitrogen-limiting conditions for 168 h. The transcriptional analyses of 13 putative genes involved in glycogen metabolism demonstrated that high level of glycogen production in nitrogen-starvation environments could be elucidated by elevated carbon flow towards glycogen synthesis, as well as increased expression levels of glycogen synthesis genes.

Sucrose is a natural product synthesized by cyanobacteria and can be accumulated intracellularly in a large amount (300 mM) [110]. A sucrose/proton symporter, i.e., sucrose permease (CscB) was introduced in 7942 enabling sucrose transport across the cell membrane [16]. Results revealed that knocking out of glycogen synthesis accompanied by the expression of CscB caused a significant amount of carbon flux towards sucrose pathway resulting in 2.7 g/L sucrose accumulation in engineered 7942 strain. Similarly, 140 mg/L of sucrose titer was recorded in a genetically engineered PCC 6803 strain by the overexpression of sucrose biosynthesizing genes and inactivating glucosylglycerol formation genes after 10 days’ incubation [111]. D-mannitol is a sugar alcohol with widespread applications in the food, pharmaceutical, and medical industries. Two enzymes namely mannitol-1-phosphate dehydrogenase and mannitol-1-phosphatase (Mlp) from *E. coli*, and the protozoan *Eimeria tenella* were expressed in 7002 strain for the biosynthesis of mannitol from sole CO_2_. The glycogen storage pathway was also disrupted to improve mannitol titer further. Notably, the engineered strain produced maximal 1.1 g/L of titer with a productivity of 0.15 g/L/d [112].

## 5. Concluding Remarks and Future Perspectives

Recent developments of metabolic engineering and synthetic biology based sophisticated and state-of-the-art tools, in the last two decades, have presented noteworthy progress in making cyanobacteria as a promising photosynthetic platform for the manufacturing of biofuels, and many commodity chemicals by fine-tuning cyanobacterial metabolism. Despite numerous successful proof-of-concept reports, however, limited work is currently being carried out to scale-up this technology. Since the commercial realization lies in the titer, productivity, and steadiness, the attributes that can only be achieved by genetically engineered cyanobacterial strains at the industrial level. This scenario quests the experimental determination of the theoretical production rates. Many concerted research efforts are required to direct or redirect a significant percentage of the fixed carbon towards the target products. In addition to the upgrade of low-cost bioreactors or open ponds infrastructure, the resulting products harvesting technology also needs to be established. Notwithstanding many challenges to the commercial feasibility of cyanobacterial chassis, the distinct perspective of these photosynthetic microorganisms is attracting continuous metabolic engineer’s interest as a green and sustainable production system.

## Figures and Tables

**Table 1:** List of industrially important chemicals produced by cyanobacterial strains, engineering strategies used, and cultivation conditions.

Host Strain	Engineering Strategies	Growth Conditions	Chemicals	Production (mg/L)	Refs
*S. elongatus* UTEX 2973	*ΔM744_RS12430:P lac - cscB -Cmr*	38 °C, 3% CO_2_, 250 μE m^−2^ s^−1^ light, 150 mM NaCl	Sucrose	35.5/h	Song, et al. [15]
*S. elongatus* PCC 7942	*cscB*- ΔInvA – ΔGlgC-*CMr*	35°C, 2% CO_2_, 65 μE m^−2^ s^−1^ light, 150 mM NaCl	Sucrose	36.1/h	Ducat et al. [16]
*S. elongatus* PCC 7942	*CscB* overexpression	32 °C, 2% CO_2_, ~80 μE m^−2^s^−1^ PAR	Sucrose	28.3/d	Weiss et al. [17]
*Synechocystis* sp. PCC6803	slr9394: Kan Prbc pdc and slr1192 slr0168: Omega Prbc pdc and slr1192	32 °C, 5% CO_2_, 100 μE m^−2^ s^−1^ light,	Ethanol	212/d	Gao et al. [18]
*S. elongatus* PCC 7942	NSI: Bb1s-dxs-idi-ispA NSII: k- P_cpcB1-_cpcB1·SF·SQS NSIII:c-P_cpcB1-_cpcB1·SF·SQS	30 °C, 5% CO_2_, 100 μE m^−2^ s^−1^ light, 10 mM MOPS	Squalene	7.08/OD_730_	Choi et al. [19]
*Synechococcus* sp. PCC 7002		30 °C, 2% CO_2_, 600 μmol photons m^−2^ s^−1^	Glycogen	3500	Aikawa et al. [20]
*Synechocystis* sp. PCC6803	5′-NS P_trc10_-lims (Ms)–ter-km^R^- 3′-NS	30 °C, 2% CO_2_, 50 μmol photons m^−2^ s^−1^	Limonene	6.7	Lin et al. [21]
*S. elongatus* PCC 7942	NSI:P_trc10_- *ls*	30 °C, 5% CO_2_, 100 μE m^−2^ s^−1^ light, 10 mM N-[Tris(hydroxymethyl)methyl]-2-aminoethanesulfonic acid	Limonene	5	Wang et al. [22]
*Synechococcus* sp. PCC 7002	NSI:ΔglgC:LS	37 °C, 1% CO_2_, 250 μmol photons m^−2^ s^−1^	Limonene	4	Davies et al. [23]
*Synechococcus* sp. PCC 7002	ΔSYNPCC7002_A2842:P_tetO2_-DHDPS-aacC1ΔSYNPCC7002_A2542:P_clac94_-ybjE-aphII	37 °C, 1% CO_2_ and 200 µmol photons m^−2^ s^−1^	Lysine	400	Korosh et al. [24]
*Synechocystis* sp. PCC6803	pEEK2-P*trc_core_*- *kivd*- *ADHΔddh*	37 °C, 50 mM NaHCO_3_ and 50 µmol photons m^−2^ s^−1^	Isobutanol	600	Miao et al. [25]
*S. elongatus* PCC 7942	AL257+NSIII:*lacI^q^; Ptrc: alsD-alsS-adh; gent^R^ + NSI:lacI^q^; Ptrc: galP-zwf-gnd; spec^R^ +cp12: lacI^q^; Ptrc: prk-rbcLXS; kan^R^*	30 °C, glucose (10 or 15 g/L), 50 mM NaHCO_3_, 30 μmol photons·m^−2^ s^−1^	2,3-butanediol	12,600	Kanno et al. [26]

**Table 2:** Growth of engineered cyanobacterial strains under different stress conditions and growth system.

Strain	Genotype/Growth	Stress Conditions	Target	Growth System	Results	Refs.
*Spirulina subsalsa*		Industrial wastewater (25%)	Protein	batch reactors	166.20 mg L^−1^d^−1^	Jiang et al. [74]
		Industrial wastewater (25%)	Lipid		64.23 mg L^−1^d^−1^	
		Industrial wastewater (50%)	Carbohydrates		48.98 mg L^−1^d^−1^	
*Synechocystis* sp. PCC 6803	30 °C, 1% (v/v) CO_2_, 50–70 μmol photons m^−2^ s^−1^	Artificial Sea water + Nitrogen + Phosphorus	Glycogen	Closed		Iijima et al. [75]
		Artificial Sea water + NPHEPHES media				
		BGG-11 media				
*Synechocystis* sp. PCC 6803	28 °C, 150 μmol photons m^−2^ s^−1^	pH-7.5	Growth	Continuous culture	12.1 mg L^−1^d^−1^	Touloupakis et al. [76]
		pH-8.5			11.7 mg L^−1^d^−1^	
		pH-9.5			11.8 mg L^−1^d^−1^	
		pH-10.0			11.5 mg L^−1^d^−1^	
		pH-10.5			10.6 mg L^−1^d^−1^	
		pH-11.0			8.2 mg L^−1^d^−1^	
*Synechocystis* Syn-HZ24		pH-11+ NaCl (300mM)	Ethanol	Closed	0.9 g/L	Zhu et al. [77]
